# Post‐hydroquinone era: Decoding shifting trends in skin‐lightening ingredient searches

**DOI:** 10.1111/jocd.16501

**Published:** 2024-07-26

**Authors:** Aarushi K. Parikh, Temitayo A. Ogunleye

**Affiliations:** ^1^ Rutgers Robert Wood Johnson Medical School New Brunswick New Jersey USA; ^2^ Department of Dermatology Perelman School of Medicine at the University of Pennsylvania Philadelphia Pennsylvania USA

To the Editor:

In March 2020, the Coronavirus Aid, Relief, and Economic Security (CARES) Act was enacted in response to the COVID‐19 pandemic. This legislation also incorporated the OTC Drug Monograph Reform Act, introducing substantial alterations to the categorization of specific over‐the‐counter (OTC) drugs, including hydroquinone.^1^

Previous categorizations included Category I: GRASE and not misbranded; Category II: not GRASE; and Category III: lacking sufficient data on safety and efficacy to permit classification. Hydroquinone was uncategorized for years; however, as part of the CARES Act, hydroquinone products were finally classified as Category II. Therefore, all hydroquinone products were removed from the OTC market on September 23, 2020.

In this study, we evaluated the Google Trends of popular skin lightening and bleaching ingredients and changes in their search volumes that may have been influenced by the OTC removal of hydroquinone.

Google Trends offers anonymized and publicly accessible data on search frequency within Google search engines. It presents search volumes in normalized Search Volume Indices (SVIs) ranging from 0 to 100. These indices are based on the proportion of total searches within a specified time period and location. Monthly SVIs in the United States of the following skin ingredients in common topical lightening/bleaching products were obtained from January 1, 2012 to December 31, 2023: hydroquinone, niacinamide, kojic acid, glutathione, and tranexamic acid. We excluded ingredients with limited SVI data such as azelaic acid, along with Vitamin C, given its prevalent oral use. The data was then analyzed for peaks and troughs of SVI. SVIs across the 50 states of the U.S. were also obtained for this time period and evaluated.

In order of greatest SVI to least, tranexamic acid exhibited the highest proportional search volume with a reported 5631 SVI, followed by glutathione (4066), hydroquinone (2415), niacinamide (3857), and kojic acid (794). General search trends for hydroquinone, niacinamide, kojic acid, glutathione, and tranexamic acid exhibit notable peaks during the spring and summer months in the Western Hemisphere, specifically from April to August. Higher percent increases for many of the ingredients occurred around March and April 2020, especially for hydroquinone, niacinamide, and kojic acid. The states with the greatest SVIs for these ingredients included: Hawaii, California, New York, Maryland, Georgia, New Jersey, Virginia, Florida, Louisiana, Washington, Nevada, Arizona, Alaska, Texas, Delaware, and Colorado.

The removal of hydroquinone from OTC products has elicited a significant shift in the active ingredients in skin‐lightening products and increased consumer interest for alternative agents.

States exhibiting increased SVI tended to boast greater racial and ethnic diversity. Seasonal patterns seen in the data may also be due to exacerbation of hyperpigmentation during summer months and/or states with sunnier climates.

Peak increases in SVI in Spring 2020 coincide with the announcement of hydroquinone removal from OTC products and an increased usage of tranexamic acid, niacinamide, and kojic acid in the absence of hydroquinone. Additionally, the unprecedented free time afforded by COVID‐19 lockdowns may have led individuals to turn to online platforms and social media for skincare information.

While the removal of OTC hydroquinone is not a significant clinical concern, data supports that patients may seek alternative skin‐lightening agents.

One limitation of the study is that many of these ingredients are also used for other purposes. The increased SVIs may be an indication of their other applications.

In conclusion, this analysis indicates underscoring a growing consumer interest in alternative skin‐lightening solutions following the enactment of the CARES Act and the subsequent removal of hydroquinone from OTC products. This shift necessitates careful consideration of their variable efficacy and safety profiles, underscoring the importance of patient education and ongoing clinical monitoring.

## AUTHOR CONTRIBUTIONS

Conceptualization, A.P.; methodology, A.P. and T.O; data curation, A.P.; writing—original draft preparation, A.P.; writing—review and editing, A.P. and T.O.; supervision, T.O. All authors have read and agreed to the published version of the manuscript.

## CONFLICT OF INTEREST STATEMENT

All authors have no conflicts of interest to disclose. No funding was obtained for this project.

## ETHICS STATEMENT

Not applicable.

## Data Availability

The data that support the findings of this study are available in Google Trends at https://trends.google.com/trends/.
